# A label-free microLC–SWATH-MS methodology with immunoaffinity depletion of highly abundant serum proteins for quantitative proteomic comparison of fresh-frozen human normal breast tissue and tumor clinical specimens

**DOI:** 10.3389/fmolb.2026.1739472

**Published:** 2026-02-19

**Authors:** Katarzyna Macur, Aleksandra E. Bogucka, Anna Fel-Tukalska, Jarosław Skokowski, Stanisław Ołdziej, Paulina Czaplewska

**Affiliations:** 1 Laboratory of Mass Spectrometry, Core Facility Laboratories, Intercollegiate Faculty of Biotechnology University of Gdańsk and Medical University of Gdańsk, Gdańsk, Poland; 2 Department of Physiology, Medical University of Gdańsk, Gdańsk, Poland; 3 Laboratory of Biopolymer Structure, Intercollegiate Faculty of Biotechnology University of Gdańsk and Medical University of Gdańsk, Gdańsk, Poland; 4 Faculty of Medicine, Academy of Applied Medical and Social Sciences – AMiSNS (Akademia Medycznych i Społecznych Nauk Stosowanych), Elbląg, Poland; 5 Clinical Department of General Surgery and Surgical Oncology, “Saint Wojciech” Hospital, “Copernicus” Health Center, Gdańsk, Poland

**Keywords:** breast cancer, breast tissue, breast tumor, extracellular matrix proteins, immunoaffinity depletion of highly abundant proteins, microLC, quantitative proteomics, SWATH-MS

## Abstract

**Background:**

Mass spectrometry (MS)-based proteomics can provide deep insights into protein-driven molecular processes and signaling pathways in breast cancer, thereby contributing to improvements in disease diagnosis, treatment, and prevention. This study focuses on the development of a label-free quantitative proteomic profiling approach for the analysis of fresh-frozen human normal breast tissue (BTIS) and breast tumor (BTUM) samples.

**Methods:**

A pilot set of BTIS and BTUM samples obtained from eight patients diagnosed with luminal B (Lum B) or triple-negative breast cancer (TNBC) was analyzed using micro-liquid chromatography coupled to tandem mass spectrometry (microLC–MS/MS) in a data-independent acquisition sequential windowed acquisition of all theoretical fragment ion spectra (SWATH) mode. To expand proteome coverage during SWATH data extraction, an experimental spectral ion library was generated from the MS/MS spectra of a pooled sample comprising aliquots from all analyzed BTIS and BTUM samples. To expand the spectral library, the pooled sample was immunodepleted of the 14 most abundant serum proteins, enabling deeper proteome coverage.

**Results:**

A total of 562 proteins were identified at a false discovery rate (FDR) of <1%, of which 299 were successfully quantified across all samples. Among these, 158 proteins showed statistically significant differences (p < 0.05) between breast tumor and normal breast tissue samples, including 59 proteins that were upregulated and 23 that were downregulated by at least 1.5-fold. Functional enrichment analysis revealed that the quantified proteins were associated with cellular structures and compartments relevant to breast cancer biology, such as the extracellular matrix (ECM), extracellular exosomes, and nucleosomes. These proteins were also involved in biological processes implicated in disease development and progression, including ECM organization, focal adhesion, mRNA splicing via the spliceosome, interleukin-12-mediated signaling, platelet activation, and metabolic pathways related to amino acid metabolism and gluconeogenesis/glycolysis.

**Conclusion:**

This proof-of-concept study demonstrates that the developed microLC–SWATH-MS approach, combined with a custom spectral library generated from pooled breast tissue and tumor samples immunoaffinity-depleted of 14 high-abundance serum proteins, enables robust and high-throughput proteomic profiling of breast tissue and tumors. Further expansion of high-quality spectral libraries may enhance proteome coverage and improve the clinical applicability of this approach. While the methodology supports the discovery of candidate biomarkers and therapeutic targets relevant to translational research and precision oncology, the biological conclusions drawn from this study should be interpreted with caution due to the limited sample size. Validation in larger patient cohorts using orthogonal methods will be required to confirm the potential clinical utility of the identified proteins.

## 1 Introduction

Breast cancer has been intensively investigated for decades, resulting in the development of novel diagnostic tools and therapeutic strategies that have improved disease control, prolonged patient survival, and enhanced quality of life (Burguin et al., 2021; Burstein, 2022). The integration of molecular testing with histopathological examination has enabled more accurate tumor classification, prognosis assessment, and therapy selection (Cardoso et al., 2019; Sun et al., 2021). Nevertheless, breast cancer remains a major clinical challenge and is still the most frequently diagnosed cancer and the leading cause of cancer-related mortality among women worldwide (Burstein, 2022).

Breast cancer is a highly heterogeneous disease, comprising multiple molecular subtypes that differ in clinical behavior, treatment options, and prognosis. Among the four intrinsic breast cancer subtypes, this study focuses on the proteomic profiles of two: luminal B–HER2-positive (Lum B–HER2(+)) and triple-negative breast cancer (TNBC). The Lum B–HER2(+) subtype is immunohistochemically characterized by positivity for estrogen receptor (ER), progesterone receptor (PR), and human epidermal growth factor receptor 2 (HER2). Although Lum B tumors are associated with an unfavorable prognosis, their molecular characteristics allow treatment with chemotherapy, endocrine therapy, and anti-HER2 agents. In contrast, TNBCs are defined by the absence of ER, PR, and HER2 expression, and their systemic treatment options are largely limited to chemotherapy (Cardoso et al., 2019; Burguin et al., 2021). Among breast cancer subtypes, TNBCs pose particular clinical challenges due to their aggressive behavior, poorer prognosis, and typically larger tumor size and higher histological grade (Burstein, 2022).

Further progress in breast cancer diagnosis and treatment, therefore, requires a comprehensive understanding of the molecular events underlying the transformation of normal breast tissue into distinct tumor types, particularly TNBCs. Although genetic assays, such as Prosigna (PAM50), have significantly improved breast cancer classification, numerous studies have demonstrated a weak correlation between transcript and protein abundance for many genes, underscoring the importance of studying gene products at the protein level (Bouchal et al., 2019; Johansson et al., 2019). This need can be addressed by mass spectrometry (MS)-based proteomic approaches, which enable comprehensive profiling of protein expression in biological samples. Ideally, such approaches should be robust, time- and cost-efficient, and capable of delivering accurate and reproducible measurements across large clinical cohorts.

To meet these requirements, a label-free data-independent acquisition (DIA) MS strategy, known as sequential windowed acquisition of all theoretical fragment ion spectra (SWATH), was developed. Unlike conventional data-dependent acquisition (DDA), in which only selected precursor ions are fragmented, data-independent acquisition (DIA) fragments all ions within predefined mass windows, enabling the detection of nearly all ionizable components in a sample. Protein identification is achieved by comparing high-quality MS/MS spectral libraries, while targeted data extraction enables accurate protein quantification based on extracted ion chromatograms, analogous to selected reaction monitoring (SRM), the gold standard for quantitative MS analysis. Owing to these features, SWATH-MS is widely regarded as a promising approach for generating reliable proteomic datasets from large clinical sample collections and supporting biomarker discovery efforts. Such proteomic maps could complement biobank specimen data and provide deeper insight into disease-specific molecular alterations, including those associated with breast cancer (Liu et al., 2013; Sajic et al., 2015).

Although library-free DIA approaches are continuously being developed (Ting et al., 2017; Demichev et al., 2020; Zhang et al., 2023), spectral libraries remain the classical and widely used means of extracting information from DIA datasets. Spectral libraries define the proteins that can be reliably detected in SWATH-acquired samples and are most commonly generated experimentally from DDA analyses of the samples of interest (Ludwig et al., 2018). To increase proteome coverage during library generation, samples are frequently fractionated prior to LC–MS/MS analysis using methods such as SDS-PAGE, high-pH reversed-phase fractionation, strong anion exchange (SAX), isoelectric focusing (IEF), depletion of high-abundance proteins (HAPs), enrichment of low-abundance proteins (LAPs), or combinations thereof (Chen et al., 2017; Zhu et al., 2018; Kaur et al., 2021). While extensive fractionation strategies, such as high-resolution isoelectric focusing (HiRIEF), provide deep proteome coverage, they are labor-intensive and require substantial resources for both sample preparation and MS analysis (Branca et al., 2014). Therefore, in this study, we aimed to enhance proteome coverage while minimizing experimental complexity. To this end, we incorporated LC–MS/MS data from pooled breast tissue and tumor samples immunodepleted of 14 high-abundance serum proteins into the spectral library, following the approach proposed by Kaur et al. (2021).

Proteomic samples are typically separated by nano-liquid chromatography (nLC) prior to MS/MS detection. Compared with analytical LC, which operates at flow rates of 0.5–2 mL/min, nLC uses flow rates of 100–1,000 nL/min, delivering highly concentrated analyte droplets to the electrospray ion source and thereby improving ionization efficiency and detection sensitivity. Despite these advantages, nLC systems often suffer from limitations, including frequent clogging, leakage, long analysis times, and reduced robustness, particularly in large-scale studies. To overcome these challenges and enable high-throughput quantitative proteomics in large clinical cohorts, microflow LC (microLC) has emerged as an attractive alternative. MS-based proteomic analyses using microLC have demonstrated satisfactory proteome coverage while offering improved robustness, ease of operation, and higher throughput. When combined with SWATH data acquisition and streamlined sample preparation, microLC-based workflows represent a promising strategy for large-scale biomarker studies (Vowinckel et al., 2018; Bian et al., 2020).

Accordingly, the focus of this study is methodological development. We propose and evaluate a strategy for proteomic biomarker research in clinical breast tumor and tissue samples based on low-maintenance microLC separation with fast gradients combined with high-throughput SWATH-MS acquisition. As a proof of concept, we applied this workflow to a small set of human clinical samples comprising fresh-frozen breast tumors and matched adjacent normal breast tissue from eight patients with Lum B and TNBC subtypes. SWATH data were analyzed using a spectral library constructed from DDA-acquired MS/MS spectra of pooled breast tissue and tumor protein extracts, generated with and without immunodepletion of the 14 most abundant human serum proteins. In addition, we performed preliminary comparisons of proteomic profiles between tumor and normal breast tissue and between TNBC and Lum B samples. These results demonstrate the utility of our methodology for protein quantification and pathway-level analysis relevant to breast cancer biology. However, due to the limited sample size and consequent low statistical power, the findings require orthogonal validation in larger, independent cohorts.

## 2 Materials and methods

### 2.1 Clinical samples

Fresh-frozen human normal breast tissue (BTIS) and breast tumor (BTUM) samples were obtained from eight patients undergoing mastectomy for breast cancer. Samples were acquired from the Central Biobank of Tissue and Genetic Material of the Medical University of Gdańsk (MUG, Poland) and stored at −70 °C until analysis. For each patient, paired tissue sections were collected, consisting of one breast tumor sample (C) and one normal breast tissue sample (P) taken at a distance from the tumor margin (sample identifiers: 5, 7, 8, 11, 12, 15, 19, and 20).

The use of human tissue samples in this study was approved by the independent local Ethics Committee of MUG (approval no. NKEBN/781/2005MUG). Prior to proteomic analysis, a pathologist examined all tissues to determine tumor stage according to the TNM Classification of Malignant Tumors, Ninth Edition (Brierley et al., 2016). Estrogen receptor, progesterone receptor, and HER2 receptor status were also assessed as part of routine pathological evaluation. A detailed description of the analyzed samples is provided in Supplementary Table S1 (Supplementary Material).

### 2.2 Sample preparation

BTIS and BTUM samples were thawed, and residual blood was removed by washing with phosphate-buffered saline (PBS; Sigma-Aldrich, Darmstadt, Germany). After weighing and sectioning, the samples were manually homogenized using a Dounce homogenizer. Proteins were extracted in 400 µL of lysis buffer containing 1% Triton X-100, 2 M thiourea, 5 M urea, 50 mM Tris-HCl (pH 7.5), 150 mM NaCl, 2% CHAPS, 0.2% ampholytes (pH 3–10), 1.5% SB3-10, 1 mM EDTA, and 1 mM dithiothreitol (DTT). Homogenates were subjected to double sonication for 10 min at 4 °C (with a 5 min break between cycles), followed by centrifugation at 14,000 × g (12,283 rpm) for 20 min at 4 °C using a Microcentrifuge 5415R (Eppendorf, Hamburg, Germany). Supernatants were transferred to low protein-binding tubes (Eppendorf), mixed with 1.6 mL of ice-cold acetone, and incubated at −20 °C for 5 h to precipitate proteins. Samples were then centrifuged (14,000 × g, 20 min, 4 °C), and the supernatants were discarded. Protein pellets were dried by vacuum evaporation (Vacufuge, Eppendorf) and resuspended in 100 µL of freshly prepared 50 mM ammonium bicarbonate (NH_4_HCO_3_). Total protein concentrations were determined spectrophotometrically using a NanoDrop ND-1000 (Thermo Fisher Scientific, Wilmington, DE, United States). The average protein concentration was 13.96 mg/mL for BTIS extracts and 11.44 mg/mL for BTUM extracts. The average extraction yield was 29.97 µg of protein per mg of tissue sample. Protein extracts were stored at −70 °C until further analysis. All procedures were performed on ice, as previously described (Macur et al., 2019).

Pooled extracts were prepared separately for BTIS and BTUM samples by combining 20 µL aliquots from each sample. The pooled extracts were diluted 1:50 in 50 mM NH_4_HCO_3_ for direct enzymatic digestion. For a subset of pooled BTIS and BTUM samples, buffer exchange to a MARS-14-compatible buffer was performed by ultrafiltration using a 3-kDa Amicon filter (Merck) followed by filtration through a 0.22-µm cellulose acetate membrane (Agilent Technologies). Immunodepletion of the 14 most abundant human serum proteins was carried out using the MARS-14 Spin Cartridge (Multiple Affinity Removal Spin Cartridge Human 14, Agilent Technologies) according to the manufacturer’s instructions. Following depletion, the buffer was exchanged to a trypsin-compatible buffer using a 3-kDa Amicon filter. Two categories of samples were prepared.

1. Pooled BTIS and BTUM samples for data-dependent acquisition (DDA) microLC–MS/MS analysis used for spectral library generation, including (a) MARS-14-depleted fractions enriched in low-abundance proteins, (b) MARS-14-retained fractions containing high-abundance proteins and co-precipitating proteins, and (c) undepleted pooled BTIS and BTUM samples.
2. Individual BTIS and BTUM samples from each patient, analyzed by data-independent acquisition (DIA) microLC–MS/MS in SWATH mode for quantitative analysis.

For the following steps, protein extract amounts equal to 100 µg of protein were used. All samples were reduced with 10 mM DTT at 56 °C for 30 min, followed by alkylation with 20 mM iodoacetamide in the dark at room temperature for 30 min. Proteins were digested with trypsin at an enzyme-to-substrate ratio of 1:50 at 37 °C for 19 h. Digestion was terminated by acidification to pH 3 using 5% formic acid (FA) in acetonitrile (ACN). Samples were dried in a SpeedVac and reconstituted in 50 µL of 0.5% trifluoroacetic acid in water. The peptide concentrations were measured spectrophotometrically in the obtained digests using NanoDrop ND-100. Then, the digest amounts equal to 10 µg of peptides were desalted using Pierce C18 Spin Tips (Thermo Fisher Scientific). Peptides were eluted sequentially with 30%, 50%, and 80% ACN containing 0.1% FA, pooled, evaporated to dryness, reconstituted in 30 µL of 50% ACN/0.1% FA, and subjected to microLC–MS/MS analysis.

### 2.3 MicroLC–MS/MS analysis

Chromatographic separation was performed using an Ekspert MicroLC 200 system (Eksigent). Five microliters of each sample were injected using a CTC PAL autosampler and separated on a ChromXP C18CL microLC column (150 × 0.3 mm, 3 μm, 120 Å; Eksigent). Peptides were eluted using a linear gradient from 10% to 90% mobile phase B over 30 min at a flow rate of 10 μL/min (mobile phase A: 0.1% FA in water; mobile phase B: 0.1% FA in ACN).

Mass spectrometric detection was performed in positive-ion mode on a TripleTOF® 5600+ mass spectrometer equipped with a DuoSpray ion source and electrospray ionization (SCIEX). Data acquisition was controlled using Analyst TF 1.7.1 software (SCIEX).

For spectral library generation, DDA shotgun MS experiments were performed with the following settings: TOF MS survey scans acquired over an m/z range of 100–2,000 for 50 ms, followed by selection of the top 10 precursor ions (charge states +2 to +5) for collision-induced dissociation. Rolling collision energy was applied, and precursor ions were excluded from reselection for 5 s after two occurrences. Product ion spectra were acquired over an m/z range of 100–2,000 for 40 m, resulting in a total cycle time of 1.11 s. This duty cycle was optimized to match typical chromatographic peak widths, ensuring sufficient data points for accurate peak area determination and quantification.

Quantitative analysis of individual patient samples was performed using SWATH-MS in data-independent acquisition mode. SWATH-MS experiments were conducted using 25 overlapping isolation windows (25 Da width) covering a precursor m/z range of 400–1,000. Product ion spectra were acquired over an m/z range of 100–2,000. Collision energies were calculated for charge states +2 to +5 and centered on each isolation window with a spread of 2. MS1 survey scans were acquired in high-sensitivity mode for 50 ms, followed by 40 ms of high-sensitivity product ion scans, yielding a total cycle time of 1.11 s.

### 2.4 Data processing

Protein database searches of DDA-MS data were performed using ProteinPilot 4.5 software (SCIEX) with the Paragon algorithm against the Swiss-Prot *Homo sapiens* database (version 31.07.2017, 20,214 entries). Searches were conducted with automated false discovery rate (FDR) estimation using the following parameters: TripleTOF 5600 instrument, trypsin digestion, iodoacetamide alkylation, biological modifications enabled, “thorough ID” search effort, and a detected protein confidence threshold >10%. Three database searches were performed: (1) pooled control breast tissue samples (undepleted and MARS-14-depleted), (2) pooled breast tumor samples (undepleted and MARS-14-depleted), and (3) a combined search including all samples, which was used to generate the spectral library. Protein identifications with an FDR <1% were considered valid. The resulting group file was imported into MS/MS All with SWATH Acquisition MicroApp 2.01 in PeakView 2.2 (SCIEX) to automatically generate the spectral library, allowing modified peptides and excluding shared peptides. A total of 144 SWATH-MS files from individual clinical samples were processed using the generated spectral library. Data extraction parameters included a maximum of six transitions per peptide, peptide confidence ≥95%, peptide FDR <1%, an extraction window of 10 min, and an extracted ion chromatogram (XIC) width of 75 ppm. For each protein, two to six unique peptides with the highest scores were selected for quantification. Proteins meeting these criteria were considered successfully quantified.

All mass spectrometry proteomics data have been deposited in the ProteomeXchange Consortium (Deutsch et al., 2023) via the PRIDE (Vizcaíno et al., 2016) partner repository under the dataset identifier PXD008408. Quantitative data were imported into MarkerView 1.2.1.1 (SCIEX) and normalized using the total area sums (TAS) method. Normalized data were further analyzed using Perseus 1.6.14 (Tyanova et al., 2016). Technical replicates were combined using median values. Samples were grouped according to tissue type (tumor vs. normal tissue) and breast cancer subtype (Lum B vs. TNBC). Group medians were calculated to determine fold changes. Data were log_2_-transformed prior to statistical testing. Paired t-tests with permutation-based FDR correction were performed to compare tumor and normal tissue samples using: (1) all eight patients, (2) only Lum B patients (n = 3), and (3) only TNBC patients (n = 5). Differences were considered statistically significant at q < 0.05. Protein interaction networks were generated using Cytoscape 3.8.2 (Shannon et al., 2003) with data from the STRING database (Szklarczyk et al., 2023). Heatmaps and principal component analysis (PCA) plots were generated using ClustVis (Metsalu and Vilo, 2015).

## 3 Results

### 3.1 Proteins identified and quantified in fresh-frozen human breast tissue and tumor samples using microLC–SWATH-MS and high-abundance serum protein depletion for spectral library generation

Protein extracts from fresh-frozen breast tumor samples and matched adjacent normal breast tissue obtained from eight patients were analyzed using a microLC–SWATH-MS workflow to generate proteome profiles. To construct a spectral library suitable for SWATH data extraction, pooled protein extracts from all normal breast tissue and tumor samples were prepared both with and without immunoaffinity depletion of high-abundance proteins (HAPs) using the MARS system. The 14 proteins targeted by this depletion strategy account for approximately 94% of the total human plasma proteome. Both the immunodepleted fractions and the corresponding HAP-containing fractions were analyzed and included in the database search for spectral library construction, thereby maximizing the probability of identifying and quantifying low-abundance proteins using a comprehensive commercial solution (Jankovska et al., 2019).

The pooled samples were analyzed in data-dependent acquisition (DDA) mode. Database searching of MS/MS spectra acquired in DDA microLC–MS/MS analyses identified 280 and 522 proteins (FDR <1%) in pooled normal breast tissue and tumor samples, respectively. In total, 547 distinct proteins were detected across both pooled sample types. When considering high-confidence identifications, defined as proteins identified by at least two unique peptides with ≥95% confidence, 199 proteins were detected in the pooled normal breast tissue sample, and 364 proteins were detected in the pooled tumor sample, yielding 411 unique proteins in total (Supplementary Tables S2 and S3, Supplementary Material). The final spectral library generated for normal and cancerous breast tissue comprised 581 proteins, of which 405 met high-confidence identification criteria (Supplementary Table S4, Supplementary Material).

This spectral library was subsequently used to quantify proteins in individual fresh-frozen normal breast tissue and tumor samples from the eight patients using microLC–SWATH-MS data. Overall, 299 proteins were both identified and quantified across the patient samples (Supplementary Tables S6 and S7, Supplementary Material). With the exception of histone H2B type F-S, immunoglobulin gamma-1 heavy chain, and immunoglobulin lambda constant 3, all quantified proteins have previously been reported in the context of breast cancer, either in patient-derived samples or breast cancer cell lines (Pecorari et al., 2009; McKiernan et al., 2011; Böhm et al., 2012; Gautrey and Tyson-Capper, 2012; Champattanachai et al., 2013; Elsawaf et al., 2013; Chen et al., 2014; Dowling et al., 2014; Ishiba et al., 2014; Muñiz Lino et al., 2014; Fernández-Grijalva et al., 2015; Jane Scully et al., 2015; Lawrence et al., 2015; Nicolini et al., 2015; Gajbhiye et al., 2016; Storr et al., 2016; Suman et al., 2016; Fujioka et al., 2017; Keyvani-Ghamsari et al., 2017; Liu et al., 2017; Zhang et al., 2017; Fletcher et al., 2018; Johansson et al., 2019; Kurpińska et al., 2019; Li et al., 2019; Yu et al., 2019; Jang et al., 2020; Trilla-Fuertes et al., 2020; Amin et al., 2021; An et al., 2021; Zhou et al., 2021; Uddin and Wang, 2022; Wu et al., 2022; Akkoc et al., 2023; Cai et al., 2023; Fallatah et al., 2023; Han et al., 2023).

### 3.2 Differentially expressed proteins between Lum B and TNBC breast tumors and adjacent normal breast tissue

Proteomic profiling of the Lum B and TNBC breast tissue and tumor samples primarily served as a proof of concept for the developed microLC–SWATH-MS methodology. Nevertheless, to assess the utility of this approach for protein quantification and pathway-level analysis relevant to breast cancer biology, we performed a preliminary comparative analysis of proteomic profiles between tumor and normal breast tissue samples, as well as between Lum B and TNBC tumors.

Across all analyzed samples, the abundance of 158 quantified proteins differed significantly (p < 0.05) between normal breast tissue and breast tumor samples (Supplementary Table S5, Supplementary Material). Among these, 82 proteins showed at least a 1.5-fold change, with 59 proteins upregulated and 23 proteins downregulated in tumors compared with normal breast tissue. When Lum B breast tumors were compared specifically with matched adjacent normal tissue, 151 proteins showed statistically significant differences, of which 138 were upregulated, and nine were downregulated by at least 1.5-fold (Supplementary Table S5, Supplementary Material). In the TNBC group, comparison with adjacent normal breast tissue revealed 76 proteins with statistically significant changes (p < 0.05), including 29 upregulated proteins and 26 proteins downregulated by at least 1.5-fold (Supplementary Table S5, Supplementary Material).

**FIGURE 1:**
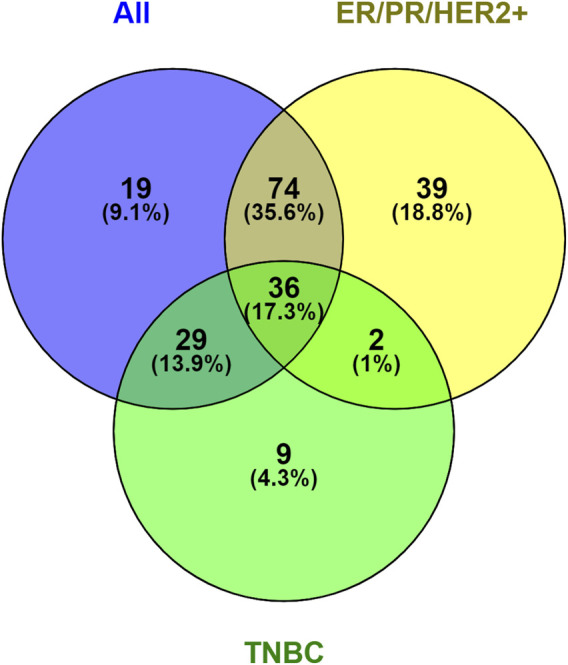
Venn diagram showing common and unique proteins that were detected and quantified with statistically significant differences between breast tumor and normal breast tissue samples across the studied groups: all tumors vs. all normal tissues, luminal B (Lum B) breast tumors vs. normal breast tissues, triple-negative breast cancer (TNBC) tumors vs. normal breast tissues, and Lum B vs. TNBC breast tumors. The Venn diagram was generated using Venny 2.1. (https://bioinfogp.cnb.csic.es/tools/venny/).

We performed a comparative analysis to determine the overlap and uniqueness of differentially expressed proteins among the following comparisons: all tumors *versus* all normal tissues, Lum B tumors *versus* normal tissue, TNBC tumors *versus* normal tissue, and Lum B *versus* TNBC tumors. The results of this analysis are summarized in a Venn diagram (Figure 1; Supplementary Table S8, Supplementary Material). Thirty-six proteins were differentially expressed across all three tumor-versus-normal comparisons. Nineteen proteins were unique to the comparison of all tumors *versus* all normal tissues, while 74 and 29 proteins overlapped with the Lum B-specific and TNBC-specific comparisons, respectively. Thirty-nine proteins were uniquely altered in Lum B tumors relative to normal tissue, whereas only nine proteins were unique to the TNBC comparison. Only two proteins, heterogeneous nuclear ribonucleoprotein A/B and protein S100-A13, were significantly differentially expressed (p < 0.05) in both Lum B and TNBC tumors compared with their respective adjacent normal tissues when these subtypes were analyzed separately (Figure 1; Supplementary Table S8, Supplementary Material).

**FIGURE 2:**
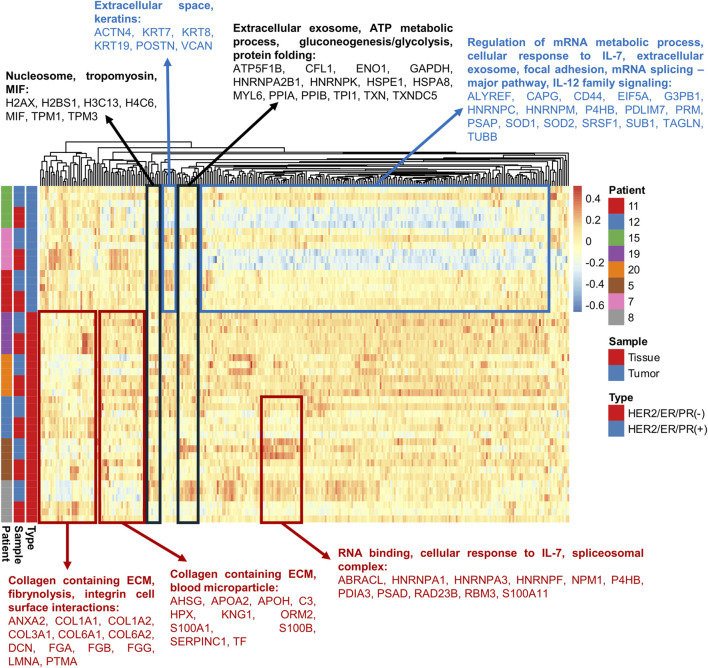
Heatmap of proteomic data for all 299 quantified proteins in breast tissue and tumor samples from eight patients. Colored frames indicate protein level changes: red for TNBC, blue for Lum B, and black for proteins altered in both Lum B and TNBC. Original values were ln(x)-transformed. Columns were centered, and vector scaling was applied. Proteins (n = 299) were clustered using correlation distance and average linkage.

The magnitude and direction of protein abundance changes varied both between tumor and normal tissue samples from the same patients and between the two investigated tumor subtypes (Lum B and TNBC) (Figure 2; Supplementary Table S5, Supplementary Material). Protein expression patterns in breast cancer are known to vary across studies and are often inconsistent. One example is decorin (DCN; P07585), an extracellular matrix proteoglycan implicated in inhibiting cancer cell proliferation and invasion. Decorin has been reported as both upregulated and downregulated in breast cancer. Oda et al. (2012) demonstrated that reduced stromal DCN expression correlates with more aggressive breast tumors, consistent with our observation of decreased DCN levels in tumor tissue compared with adjacent normal breast tissue (Supplementary Table S5, Supplementary Material). In contrast, Hosoya et al. (2021) reported increased plasma DCN levels with breast cancer progression. These discrepancies likely reflect tumor heterogeneity, differences in disease stage, biological material analyzed, and variability in sample preparation and protein extraction methods, underscoring the complexity of breast cancer biology.

**FIGURE 3:**
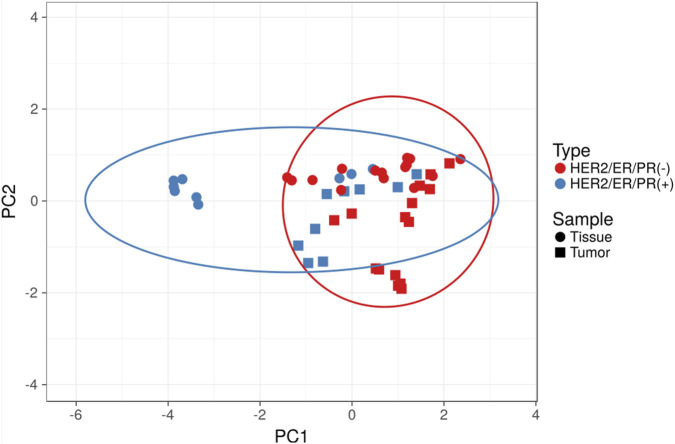
Principal component analysis (PCA) of the proteomic data acquired in this study. Original values were ln(x)-transformed, and vector scaling was applied to rows. Principal components were calculated using singular value decomposition (SVD) with imputation. The X and Y axes represent principal component 1 and principal component 2, explaining 44.2% and 10.9% of the total variance, respectively. Prediction ellipses indicate the 95% confidence interval, such that a new observation from the same group is expected to fall within the ellipse. N = 48 data points.

Hierarchical clustering based on protein abundance enabled clear separation of normal breast tissue and Lum B tumor samples for two of the three Lum B patients included in the study (Figure 2). The remaining Lum B patient displayed a distinct proteomic profile, suggesting additional molecular features not captured by current clinical classification methods. In contrast, separation between normal and tumor tissue samples was less pronounced for TNBC patients, as reflected in the corresponding heatmap (Figure 2). Principal component analysis (PCA) further demonstrated partial overlap between tumor and normal tissue samples, particularly within the TNBC group (Figure 3). These observations highlight the substantial heterogeneity of breast tumors and suggest that proteomic profiling using the developed microLC–SWATH-MS approach may contribute to more refined molecular characterization and potentially more personalized therapeutic strategies. However, the limited sample size of this proof-of-concept study likely contributed to the reduced discriminatory power observed. Therefore, future biomarker discovery studies using this strategy should include larger patient cohorts, detailed molecular tumor characterization, comprehensive clinical annotation, and validation using orthogonal analytical methods.

### 3.3 Bioinformatic analysis of proteomic data to identify biological processes, molecular functions, and cellular components

Two complementary bioinformatic analyses were performed to characterize the proteins identified and quantified in this study. First, STRING (Szklarczyk et al., 2023) was used to classify the quantified proteins according to Gene Ontology (GO) biological processes, molecular functions, and cellular components in breast tumor and adjacent normal breast tissue samples (Figure 2). Many quantified proteins were associated with collagen-containing extracellular matrix (ECM), extracellular space, blood microparticles, spliceosomal complexes, nucleosomes, tropomyosin-containing structures, macrophage migration inhibitory factor (MIF) complexes, focal adhesions, and extracellular exosomes (Figure 2). These proteins participate in biological processes known to be altered during breast cancer development and progression, including integrin-mediated cell surface interactions (Liu F. et al., 2023), fibrinolysis (Lal et al., 2013), cellular responses to interleukin-7 (Zarogoulidis et al., 2014; Sermaxhaj et al., 2022), interleukin-12 family signaling (Habiba et al., 2022), RNA binding and regulation of mRNA metabolism (Gahete et al., 2022; Cui et al., 2024), mRNA splicing via the major spliceosomal pathway (Gahete et al., 2022), ATP metabolic processes and gluconeogenesis/glycolysis (Wang and Dong, 2019), and protein folding (Brodsky, 2017) (Figure 2).

**FIGURE 4:**
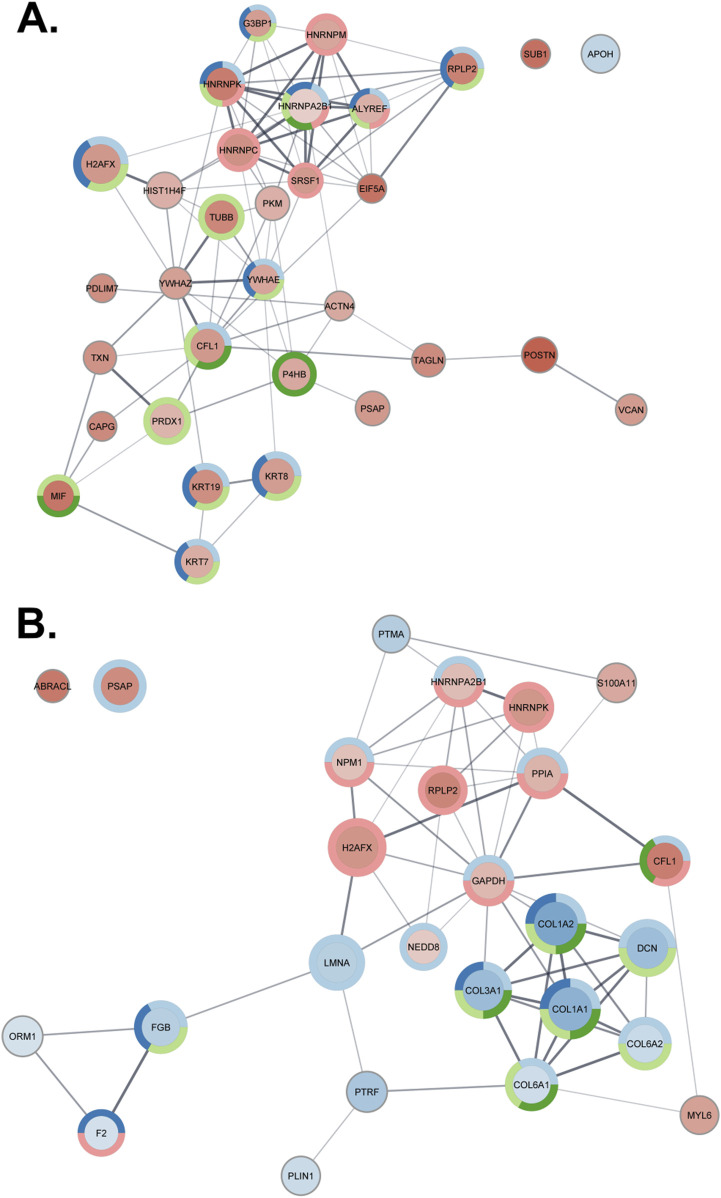
Protein interaction networks of proteins present at significantly different concentrations between tumor and healthy tissue samples (fold change >1.5, q-value <0.01). Circle size corresponds to the log_10_ of the median protein abundance in healthy tissue, while color intensity reflects the log2 fold change (red indicates higher, and blue indicates lower concentrations in tumor samples). Pie charts surrounding the circles represent functional enrichment in the Gene Ontology biological process category. **(A)** Lum B samples. The pie chart colors represent the following categories: light blue, symbiotic process; dark blue, viral process; light green, interspecies interaction between organisms; dark green, interleukin-12-mediated signaling pathway; red, mRNA splicing via the spliceosome. **(B)** TNBC samples. The pie chart colors represent the following categories: light blue, response to organic substance; dark blue, platelet activation; light green, extracellular matrix organization; dark green, response to amino acid; red, symbiotic process.

In the second analysis, proteins exhibiting at least a 1.5-fold change between breast tumors and adjacent normal breast tissue were analyzed separately for Lum B and TNBC subtypes. Interaction networks were constructed in Cytoscape (Shannon et al., 2003) to visualize enriched GO biological processes (Figure 4). Compared with normal breast tissue, Lum B [ER(+)/PR(+)/HER2(+)] tumors showed upregulation of biological processes related to symbiotic interactions, viral processes, interspecies interactions, interleukin-12-mediated signaling, and mRNA splicing via the spliceosome (Figure 4A). In contrast, TNBC [ER(−)/PR(−)/HER2(−)] tumors exhibited downregulation of processes related to ECM organization, responses to amino acids and organic substances, and platelet activation, alongside upregulation of symbiotic processes (Figure 4B). Several of these pathways have been extensively studied in the context of breast cancer, including interleukin-12 signaling (Habiba et al., 2022), mRNA splicing (Gahete et al., 2022), ECM organization (Bonnans et al., 2014; Jena and Janjanam, 2018), metabolic and stress responses (Lee and Lam, 2025), and platelet activation (Braun et al., 2021). In contrast, the involvement of biological processes such as symbiotic interactions, viral processes, and interspecies interactions remains relatively unexplored in breast cancer research (Alibek et al., 2013; Icard et al., 2014; Afzal et al., 2022).

## 4 Discussion

### 4.1 Applicability of the microLC–SWATH-MS approach with high-abundance protein immunoaffinity depletion for quantitative proteomic analysis of fresh-frozen breast tissue and tumor specimens

The number of proteins detected and quantified in the present study is lower than that reported in several large-scale breast cancer proteomics studies, including those by Lawrence et al. (2015) and Johansson et al. (2019). This discrepancy likely reflects multiple factors, foremost among them biological variability and study design. In contrast to these comprehensive analyses, which included numerous breast cancer subtypes and, in some cases, breast cancer cell lines, our study focused specifically on two clinically relevant subtypes, luminal B and TNBC, and on matched adjacent normal breast tissue obtained from the same patients.

Notably, Lawrence et al. (2015) reported the detection of several proteins exclusively in breast cancer cell lines, such as mammaglobin B (SCGB2A1), cyclin 1 (CYLC1), and proto-oncogene ROS1, which were not consistently detected in tumor tissues and therefore require further validation in patient-derived specimens. In contrast, defensin-5 (DEFA5), which was detected by Lawrence et al. (2015) only in a single cell line (SW527), was quantified in both normal and tumor tissues in our study. Similarly, anterior gradient protein two homolog (AGR2) was quantified across all samples analyzed here, whereas it was detected only in a subset of tumor samples in the Lawrence et al. (2015) dataset. Although AGR2 abundance did not differ significantly between tumor and normal tissue in our cohort, previous studies have reported elevated AGR2 expression in non-TNBC breast cancers compared with normal tissue (Guo et al., 2017). AGR2 has also been implicated in tumorigenesis and metastasis, and its secreted form is thought to promote cancer cell adhesion and metastatic spread (Salmans et al., 2013). More broadly, even large-scale proteomic studies of breast cancer only partially overlap in terms of identified and quantified proteins, including overlap with smaller-scale studies, such as those by Böhm et al. (2012) and Coumans et al. (2014). These observations underscore the biological complexity of breast cancer and highlight the need for robust, scalable methodologies capable of analyzing large clinical cohorts—an application for which the rapid and relatively simple quantitative proteomic workflow presented here may be well suited.

Beyond biological variability, the choice of analytical workflow and instrumentation substantially influences proteome coverage. Many in-depth breast cancer proteomic studies rely on nanoLC coupled with nano-electrospray ionization (nESI) MS/MS systems (Lawrence et al., 2015; Johansson et al., 2019; Djomehri et al., 2020), which offer high sensitivity due to improved ionization efficiency. However, such systems are often less robust, more maintenance-intensive, and more susceptible to technical failures than microLC–MS/MS systems equipped with conventional ESI sources. Moreover, nLC-based analyses typically require longer run times per sample, which may limit throughput in large clinical studies (Bian et al., 2020).

Proteome depth is also strongly affected by sample preparation and prefractionation strategies. For example, Johansson et al. (2019) employed extensive prefractionation using high-resolution isoelectric focusing (HiRIEF) of TMT-labeled peptides, followed by analysis of dozens of fractions per sample by nLC–MS/MS. While this approach enabled deep proteome coverage, it required extensive sample processing, numerous LC–MS/MS runs, and incorporation of isobaric labeling. Although isobaric labeling improves quantification accuracy and throughput, multiplexing capacity remains limited by tag availability (Lawrence et al., 2015), which may constrain very large clinical studies. In contrast, the label-free strategy used here imposes no inherent limits on the number of samples and avoids labeling-associated biases. Furthermore, the 30-min microLC–MS/MS gradient employed in this study, combined with minimal sample preparation, substantially reduces analysis time and cost.

Data analysis criteria further affect the number of reported proteins. In this study, emphasis was placed on robust, reproducible quantification across all samples rather than on maximizing protein identifications. Consequently, only proteins detected consistently across all samples and replicates and identified with at least two peptides were quantified. This contrasts with studies such as Lawrence et al. (2015), where many proteins were detected in only a subset of samples, and reflects our aim to prioritize quantitative reliability over proteome depth in this proof-of-concept study.

More advanced data processing strategies, such as *de novo* peptide sequencing (Vitorino et al., 2020), exploration of unexpected post-translational modifications (Kong et al., 2017), or more complex quantitative modeling (Kohler et al., 2023), could potentially increase proteome coverage and improve sample discrimination. However, given the limited sample size, we deliberately employed a streamlined and well-established workflow to demonstrate the feasibility and potential of the proposed methodology.

### 4.2 Proteomic fingerprints of breast tissue and tumor samples generated using microLC–SWATH-MS

Preliminary comparisons of proteomic profiles between breast tumor and adjacent normal tissue samples, as well as between TNBC and Lum B subtypes, identified proteins exhibiting differential abundance. Given the limited cohort size, these findings are exploratory. Nevertheless, they demonstrate the capability of the microLC–SWATH-MS approach to capture biologically relevant protein changes and the associated pathways implicated in breast cancer biology. Future studies employing larger cohorts and orthogonal validation methods will be required to assess the biomarker potential of the differentially expressed proteins identified here.

#### 4.2.1 Proteins downregulated in breast tumors relative to adjacent normal breast tissue

The proteins present at significantly lower abundance in tumor samples predominantly originated from or were associated with the extracellular matrix (ECM), including collagens, decorin, and fibrinogens. Extracellular matrix (ECM) remodeling is a hallmark of tumor progression and involves processes such as collagen degradation, deposition, cross-linking, and stiffening (Fang et al., 2014). Collagen-rich tumor microenvironments impose physical, biochemical, and immunological barriers that influence breast cancer progression and therapeutic resistance. Consequently, multiple therapeutic strategies targeting collagen have been explored in breast cancer, ranging from drug delivery systems that exploit collagen retention to approaches that modify ECM architecture, immune barriers, or specific cellular and molecular targets (Liu J. et al., 2023; Wang et al., 2025; Wu et al., 2025). In our cohort, several collagen chains (COL1A1, COL1A2, and COL3A1) were among the most strongly depleted proteins in tumor tissue, particularly in early-stage tumors (grades IIA and IIB). Although increased collagen deposition has frequently been associated with breast cancer progression (Provenzano et al., 2009; Xiong et al., 2014), breast carcinomas display marked heterogeneity in stromal composition, ranging from highly cellular tumors with minimal collagen to dense, collagen-rich lesions (Walker, 2001). Importantly, collagen organization, rather than absolute abundance, may determine whether it exerts tumor-promoting or tumor-suppressive effects (Maller et al., 2013). Furthermore, reduced collagen recovery in tumor samples may reflect altered fibril organization that limits solubilization and proteolytic digestion, consistent with the need for specialized ECM enrichment strategies in matrisome proteomics (Krasny et al., 2016). If confirmed in a sufficiently large patient cohort, our findings would suggest that the tumor-supportive properties of collagen depend more on its structural organization than on its quantity, analogous to the role of ECM stiffness. Such insights would have important implications for ECM-targeted therapies, indicating that strategies focused solely on reducing collagen abundance may be insufficient or misguided (Liu J. et al., 2023; Wang et al., 2025; Wu et al., 2025). Fibrinogen chains were also less abundant in tumor tissue in our study, in contrast to reports of increased fibrinogen levels in tumor stroma and patient sera (Dowling et al., 2014; Mei et al., 2016). However, earlier studies have shown that despite fibrinogen abundance, deregulation of coagulation and fibrinolytic pathways may prevent fibrin deposition in tumor stroma (Costantini et al., 1991), potentially reconciling these observations. Decorin, an ECM proteoglycan with tumor-suppressive functions, was markedly reduced in tumor samples. Decorin inhibits tumor cell proliferation, angiogenesis, and metastasis (Buraschi et al., 2012; Ishiba et al., 2014), and its activity can be antagonized by periostin through complex formation (Ishiba et al., 2014). Consistent with this mechanism, we observed elevated periostin levels alongside reduced decorin abundance in tumor tissue.

Additional downregulated proteins included serum albumin, immunoglobulin J chain, S100A10, cytokeratin 1, caveolae-associated protein 1 (CAVIN1), and prolactin-inducible protein (PIP). Reduced albumin levels have been associated with poorer prognosis in breast cancer (Azab et al., 2013; Liu et al., 2014). Downregulation of the J chain has been reported in other biological fluids from breast cancer patients (Böhm et al., 2012). While several S100 family members were upregulated or unchanged in our study, S100A10 was reduced, consistent with reports linking lower S100A10 expression to improved survival (Zhang et al., 2017). Although keratins are often elevated in breast tumors, cytokeratin 1 has also been implicated in tumor-suppressive processes (Blanckaert et al., 2015). Reduced CAVIN1 expression, previously observed in breast cancer, may promote cancer cell survival through increased autophagy (Bai et al., 2012; Shi et al., 2015). Finally, decreased PIP levels, particularly in early-stage tumors, are consistent with recent transcriptomic findings (Gangadharan et al., 2018).

#### 4.2.2 Proteins upregulated in breast tumors relative to adjacent normal breast tissue

Several proteins exhibited significantly increased abundance in tumor samples. Among them, peroxiredoxin 1 (PRDX1) has been linked to higher tumor grade and poorer prognosis in ER-positive breast cancer (Cha et al., 2009; O’Leary et al., 2014). Multiple histones, including H2AX, H2B, and H4, were also upregulated, reflecting altered chromatin organization and epigenetic regulation in cancer cells (Nandy et al., 2020). The increased H2AX levels observed in TNBC are consistent with previous reports (Wang et al., 2019). Nucleophosmin (NPM1), a histone chaperone involved in chromatin remodeling, was also elevated, in agreement with earlier studies (Skaar et al., 1998; Zeng et al., 2019). Thymosin β4, which influences actin dynamics and transcriptional regulation, was upregulated and has been associated with increased tumor aggressiveness (Cho, 2010; Nemolato et al., 2010).

Increased expression of ribosomal protein RPLP2 suggests altered protein synthesis and autophagy regulation in tumor cells (Chen et al., 2002; Artero-Castro et al., 2015). Additionally, stathmin and periostin, which are both linked to tumor aggressiveness, metastasis, and poor prognosis, were significantly upregulated (Morra and Moch, 2011; Baquero et al., 2012; Xu et al., 2012; Kuang et al., 2015). Stathmin destabilizes microtubules and cancer cell migration, while periostin activates integrin-mediated signaling pathways that increase invasion, angiogenesis, and metastatic spread. Increased tropomyosin alpha-3 chain (TPM3), also associated with metastatic progression, further supports these findings (Lee et al., 2012).

### 4.3 Biological pathways altered in Lum B and TNBC breast tumors

Functional enrichment analysis of proteins differing by at least 1.5-fold between tumor and normal tissue revealed subtype-specific biological processes. Upregulation of mRNA splicing via the spliceosome was observed in Lum B tumors. Aberrant splicing is a recognized feature of breast cancer and contributes to oncogenic isoform expression, therapy resistance, and disease progression (Gahete et al., 2022). Alternative splicing of ER and HER2 may be particularly relevant in Lum B tumors, influencing tumor behavior and treatment response.

Interleukin-12-mediated signaling was also upregulated in Lum B tumors. IL-12 is a potent immunomodulatory cytokine with anti-tumor activity, and increased IL-12 levels have been reported in hormone receptor-positive breast cancer patients (Vilsmaier et al., 2016; Habiba et al., 2022). Additionally, GO terms related to symbiotic, viral, and interspecies interaction processes were enriched. Although initially counterintuitive, these terms likely reflect the multifunctional nature of proteins involved in both cancer-related pathways and host–pathogen interactions. For example, PPIA and GAPDH play roles in tumor progression while also participating in viral or bacterial infection processes (Obchoei et al., 2009; Gani et al., 2021; Schmidt et al., 2021). Moreover, tumor–stroma metabolic interactions have been described as a form of commensalism that supports tumor growth (Icard et al., 2014). Viral involvement in breast cancer has been proposed but remains incompletely understood (Alibek et al., 2013; Afzal et al., 2022).

In TNBC tumors, downregulation of processes related to amino acid and organic substance response reflects metabolic reprogramming, a hallmark of cancer and a defining feature of TNBC, which relies heavily on altered glycolysis, oxidative phosphorylation, and amino acid metabolism (Wang et al., 2020; Wei et al., 2021). Downregulation of ECM organization and platelet activation pathways, both well-established contributors to tumor progression, was also observed, consistent with extensive prior literature (Robertson, 2016; Braun et al., 2021; Yu et al., 2023).

## 5 Conclusion

The microLC–SWATH-MS methodology presented here provides a robust, cost-effective, and high-throughput platform for quantitative proteomic analysis of human breast tissue and tumor samples. In this proof-of-concept study, 299 proteins were reliably quantified in fresh-frozen samples from Lum B and TNBC patients. The differentially abundant proteins identified have been previously implicated in breast cancer biology and warrant further investigation in larger cohorts and orthogonal validation.

## Data Availability

The datasets presented in this study can be found in online repositories. The names of the repository/repositories and accession number(s) can be found in the article/Supplementary Material.
